# Trichoscopic Differentiation in Alopecia: Retrospective Case Series Comparing Lichen Planopilaris, Discoid Lupus Erythematosus, and Alopecia Areata

**DOI:** 10.2196/83463

**Published:** 2025-11-20

**Authors:** Gökhan Kaya

**Affiliations:** 1 Department of Dermatology, Nizip State Hospital, Dutlu, Küme Evler, Gaziantep, 27700, Turkey, +90 532 645 0034

**Keywords:** trichoscopy, dermoscopy, alopecia areata, lichen planopilaris, discoid lupus erythematosus, cicatricial alopecia, diagnostic markers

## Abstract

This single-center retrospective case series included 28 patients with alopecia (7 with lichen planopilaris, 7 with discoid lupus erythematosus, and 14 with alopecia areata). Trichoscopic markers were systematically compared across groups. Exclamation-mark hairs and yellow dots were characteristic of alopecia areata, whereas follicular ostia loss and white scarring were confined to lichen planopilaris/discoid lupus erythematosus, providing a simple and practical distinction between nonscarring and scarring alopecias in routine practice.

## Introduction

Trichoscopy is now integral to alopecia assessment, enabling the recognition of hair‐shaft changes, peri or interfollicular alterations, and follicular opening loss to quickly separate nonscarring from scarring disease [1]. In scarring alopecias, lichen planopilaris (LPP) typically shows perifollicular scales or erythema and target-pattern blue-gray dots, whereas discoid lupus erythematosus (DLE) more often displays follicular keratotic plugs with telangiectatic or arborizing vessels; patterns can vary by phototype, underscoring the need for pragmatic rules that generalize across populations [2,3]. The misclassification between LPP and DLE is well documented, emphasizing the value of simple bedside discriminators that complement histopathology [4].

This study, therefore, aimed to compare key trichoscopic markers among LPP, DLE, and AA and to propose a concise, rule-in/rule-out approach for routine clinical care.

## Methods

### Setting and Participants

This single-center, retrospective case series was conducted at the Department of Dermatology, Nizip State Hospital, Gaziantep, Türkiye, including consecutive patients with alopecia who underwent trichoscopic evaluation: LPP (n=7), DLE (n=7), and AA (n=14). The diagnosis of AA was made based on clinical and trichoscopic criteria, whereas all LPP and DLE cases were histopathologically confirmed. Age and sex were retrieved from medical records.

### Trichoscopic Evaluation

Routine polarized dermoscopy images were reviewed using a prespecified 16-item checklist (present=1/absent=0), including perifollicular scale, erythema, and casts; blue-gray target dots; follicular ostia loss or plugs; yellow and black dots; white scar or atrophy; background erythema; arborizing vessels; interfollicular scales; exclamation-mark, broken, and lonely hairs; and tufting.

Arborizing vessels were graded by caliber (0=absent, 1=thin <50% of adjacent hair-shaft caliber, 2=thick ≥50% of adjacent hair-shaft caliber).

### Statistical Analysis

Categorical variables were summarized as n (%) and continuous variables as mean (SD). Prespecified contrasts used two-sided Fisher exact tests (α=.05): AA versus scarring alopecias (LPP+DLE) for exclamation-mark hairs, yellow dots, white scarring or atrophy, and follicular ostia loss (plus exploratory black or broken hairs), and DLE versus LPP for follicular plug, arborizing vessels (any/thick), interfollicular scale, and peripilar casts.

Odds ratios were estimated with a Haldane–Anscombe 0.5 correction when zero cells occurred. Analyses were performed using Python and SciPy.

### Ethical Considerations

The study was conducted in accordance with the Declaration of Helsinki. Ethical approval was granted by the Scientific Research Ethics Committee of Bezmialem Vakıf University, Istanbul, Türkiye (Approval No: E-54022451-050.04-122609; September 6, 2023). The protocol originally focused on AA; additional LPP and DLE cases collected under the same protocol were analyzed here as a secondary evaluation, with notification to the ethics committee. All participant data were anonymized and deidentified before analysis. Owing to the retrospective design and use of anonymized data, informed consent was not required. No compensation was offered to study participants.

## Results

**Figure 1.**
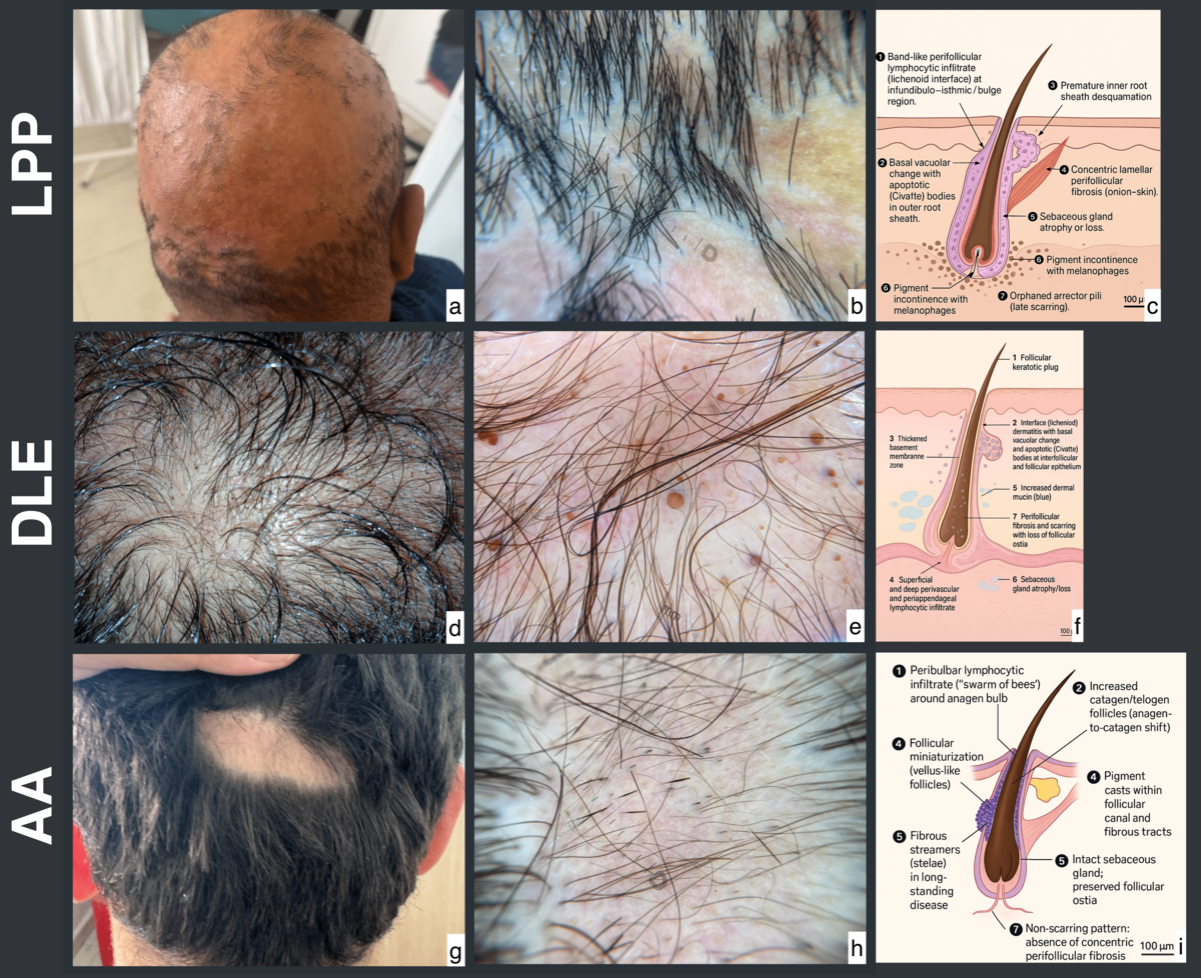
Clinical, trichoscopic, and histopathological features of LPP, DLE, and AA. (A–C) LPP: Patchy scarring alopecia with perifollicular scales or erythema and blue-gray dots; histology illustration (**C**) Perifollicular lichenoid infiltrate, lamellar fibrosis, sebaceous loss, and pigment incontinence. (D–F) DLE: Scarring alopecia with keratotic plugs, erythema, and arborizing vessels; schematic drawing (**F**) Follicular plugging, thickened basement membrane zone, dermal mucin, and perifollicular fibrosis with ostial loss. (G–I) AA: Nonscarring alopecia with yellow or black dots and exclamation-mark hairs; histopathologic illustration (**I**) Peribulbar “swarm of bees” infiltrate, follicular miniaturization, preserved sebaceous glands, and absence of concentric fibrosis. All clinical and trichoscopic images are original and de-identified; schematic histologic illustrations (**C, F, I**) were created by the authors for this figure to depict key diagnostic features. AA: alopecia areata; DLE: discoid lupus erythematosus; IRS: inner root sheath; LPP: lichen planopilaris.

Twenty-eight patients were included (7 each with LPP and DLE, and 14 with AA); age and sex distributions were comparable. Scarring signs (white scar or atrophy and follicular ostia loss) occurred exclusively in LPP/DLE and were absent in AA. Trichoscopic markers clustered in AA, most prominently exclamation-mark hairs, followed by yellow dots, whereas black dots and broken hairs were not discriminatory. Within scarring alopecias, DLE showed more follicular plugs and occasional thick arborizing vessels, while LPP showed universal perifollicular scale with more perifollicular erythema; these trends were not statistically significant given the sample size. Representative clinical, trichoscopic, and histopathological findings for each entity are illustrated in Figure 1. All estimates and exact *P* values are provided in Table 1.

**Table 1.** Demographics and key trichoscopic markers across diagnoses, with AA^a^ versus scarring statistics.

Variable	LPP^b^ (n=7)	DLE^c^ (n=7)	AA (n=14)	OR (AA vs scarring^d^)	*P* value
Age (years), mean (SD)	34.4 (10.3)	40.3 (6.6)	37.7 (6.4)	–	–
Female participant, n (%)	3 (43)	2 (29)	5 (36)	–	–
White scarring or atrophy, n (%)	7 (100)	7 (100)	0 (0)	<0.01^e^	<.001
Follicular ostia loss, n (%)	7 (100)	7 (100)	0 (0)	<0.01^e^	<.001
Exclamation-mark hairs, n (%)	0 (0)	0 (0)	11 (79)	95.29	<.001
Yellow dots, n (%)	0 (0)	2 (29)	10 (71)	15.00	.006
Black dots, n (%)	0 (0)	3 (43)	7 (50)	3.67	.237
Broken hairs, n (%)	4 (57)	2 (29)	8 (57)	1.78	.706

a AA: alopecia areata.

b LPP: lichen planopilaris.

c DLE: discoid lupus erythematosus.

d “Scarring” denotes the combined LPP+DLE group.

e Haldane–Anscombe 0.5 correction applied when any cell contained zero.

## Discussion

### Principal Findings

This study identified key trichoscopic patterns that reliably distinguish nonscarring from scarring alopecias. In this cohort, white scarring or atrophy and follicular ostia loss occurred exclusively in LPP or DLE and were absent in AA, reinforcing that the loss of follicular openings is a practical hallmark of cicatricial disease [5]. Conversely, AA showed clusters of exclamation-mark hairs and, secondarily, yellow dots; these markers also track AA activity and severity in structured trichoscopic scoring systems such as STRIAA (Severity Trichoscopy Index Alopecia Areata) and support their use as practical rule-in signs [6].

Among scarring alopecias, the findings of this study were consistent with those in previous reports [3,7,8]: DLE showed more follicular plugs and occasional thick arborizing vessels, whereas LPP consistently demonstrated perifollicular scale, frequent erythema, and characteristic target-pattern blue-gray dots [3,7,8]. Notably, lonely hair is not disease-specific and should be interpreted in context, particularly when differentiating LPP from frontal fibrosing alopecia [7].

Clinically, a succinct rule emerges: AA is favored by one or more of exclamation-mark hairs or yellow dots, whereas the combination of ostia loss and white scarring favors LPP or DLE. This aligns with stepwise diagnostic algorithms that first classify distribution, then scarring status by the presence or absence of ostia, and finally apply a short list of trichoscopic clues [9]. Comparative clinicopathologic studies also demonstrate systematic differences between DLE and LPP at the population level, providing additional context for our observations [10].

Key limitations include the small, single-center sample and limited power for LPP–DLE contrasts. Nevertheless, the direction and magnitude of the observed effects and the identified high-yield markers are consistent with contemporary systematic reviews [3] and support the external validity of the findings of this study.

### Conclusions

A minimalist trichoscopic rule effectively differentiates AA from scarring alopecias: exclamation-mark hairs or yellow dots favor AA, whereas the combination of follicular ostia loss and white scarring favors LPP or DLE. These easily recognizable cues can assist clinicians in biopsy site selection and treatment planning. Larger multicenter studies are warranted to validate these findings and refine diagnostic criteria for distinguishing LPP from DLE.

## Abbreviations

AA: alopecia areata
DLE: discoid lupus erythematosus
LPP: lichen planopilaris
STRIAA: Severity Trichoscopy Index Alopecia Areata
